# Sharing and Specificity of Co-expression Networks across 35 Human Tissues

**DOI:** 10.1371/journal.pcbi.1004220

**Published:** 2015-05-13

**Authors:** Emma Pierson, the GTEx Consortium, Daphne Koller, Alexis Battle, Sara Mostafavi

**Affiliations:** Department of Computer Science, Stanford University, Stanford, California, United States of America; Thomas Jefferson University, UNITED STATES

## Abstract

To understand the regulation of tissue-specific gene expression, the GTEx Consortium generated RNA-seq expression data for more than thirty distinct human tissues. This data provides an opportunity for deriving shared and tissue specific gene regulatory networks on the basis of co-expression between genes. However, a small number of samples are available for a majority of the tissues, and therefore statistical inference of networks in this setting is highly underpowered. To address this problem, we infer tissue-specific gene co-expression networks for 35 tissues in the GTEx dataset using a novel algorithm, GNAT, that uses a hierarchy of tissues to share data between related tissues. We show that this transfer learning approach increases the accuracy with which networks are learned. Analysis of these networks reveals that tissue-specific transcription factors are hubs that preferentially connect to genes with tissue specific functions. Additionally, we observe that genes with tissue-specific functions lie at the peripheries of our networks. We identify numerous modules enriched for Gene Ontology functions, and show that modules conserved across tissues are especially likely to have functions common to all tissues, while modules that are upregulated in a particular tissue are often instrumental to tissue-specific function. Finally, we provide a web tool, available at mostafavilab.stat.ubc.ca/GNAT, which allows exploration of gene function and regulation in a tissue-specific manner.

## Author Summary

Cells in different tissues perform very different functions with the same DNA. This requires tissue-specific gene expression and regulation; understanding this tissue-specificity is often instrumental to understanding complex diseases. Here, we use tissue-specific gene expression data to learn tissue-specific gene regulatory networks for 35 human tissues, where two genes are linked if their expression levels are correlated. Learning such networks accurately is difficult because of the large number of possible links between genes and small number of samples. We propose a novel algorithm that combats this problem by sharing data between similar tissues and show that this increases the accuracy with which networks are learned. We provide a web tool for exploring these networks, enabling users to pose diverse queries in a gene- or tissue-centric manner, and facilitating explorations into gene function and regulation.

This is a *PLOS Computational Biology* Methods paper.

## Introduction

Tissue-specificity, in which cells perform different functions despite possessing identical DNA, is achieved partially through tissue-dependent mechanisms of gene regulation, including epigenetic modification and transcriptional and post-transcriptional regulation [1–3]. These complex programs of control produce different gene expression programs across tissues, with most genes showing statistically significant differential expression [4, 5]. These differences can have significant consequences: tissue-specific genes are especially likely to be drug targets [6] and tissue-specific transcription factors are especially likely to be implicated in complex diseases [2, 7, 8]. Understanding these differences is also essential for understanding pleiotropic genes, and for interpreting studies in which genomics data can only be collected for an accessible or a proxy tissue (such as use of blood in studying psychiatric disorders [9–11]).

Tissue-specific mechanisms of control may be captured by co-expression networks, in which two genes are connected if their expression levels are correlated across a set of individuals. In such a setting, genetic or environmental differences across individuals serve as small perturbations to the underlying regulatory network, resulting in correlation between genes’ expression levels that are consistent with regulatory relationships. Co-expression networks provide insight into cellular activity as genes that are co-expressed often share common functions [12], and such networks have been widely used to study disease [13–15].

The Genotype-Tissue Expression (GTEx) consortium dataset [16] provides an opportunity to study such co-expression networks for an unprecedented number of human tissues simultaneously. However, many of the profiled tissues have fewer than a dozen samples, too few to accurately infer the tens of millions of parameters that would define a co-expression or regulatory network. One solution would be to combine all available samples and learn a single consensus network for all tissues, but this would offer no insight into tissue-specificity. On the other hand, inferring each network independently ignores tissue commonalities: tissue networks share far more links than would be expected by chance, and learning links across multiple tissues is less noisy than learning links using a single tissue, even when using the same number of total samples [12].

Here, we use a novel algorithm, GNAT (Gene Network Analysis Tool), to simultaneously construct co-expression networks for 35 distinct human tissues. Using a hierarchy which encodes tissue similarity, our approach learns a network for each tissue, encouraging tissues that are nearby in the hierarchy to have similar networks. Hierarchical transfer learning has been shown to improve power and accuracy in previous work [5, 6, 17, 18]. We propose a novel hierarchical model along with a parameter optimization method designed for large-scale data, and apply it to the GTEx data. We show that our method infers networks with higher cross-validated likelihood than networks learned on each tissue independently or a single network learned on all tissues. Our method is applicable to any dataset in which sample relationships can be described by a hierarchy—for example, multiple cancer cell lines or species in a phylogenetic tree. The complete code for our method is available as S1 Data.

We analyze the resulting networks to make several novel observations regarding principles of tissue-specificity. We propose multiple metrics for identifying genes that are important in defining tissue identity, and demonstrate that such genes are disproportionately essential genes. We show that tissue-specific transcription factors, which are central hubs in our networks, link to genes with tissue-specific functions, which in turn display higher expression levels. We identify 1,789 gene modules that are enriched for Gene Ontology functions, and show that enriched modules that are upregulated within a tissue are often instrumental to tissue function. We also show that modules which occur across tissues are especially likely to be enriched for Gene Ontology functions, and that these functions tend to be those which are essential to all tissues. The results presented here, including all the networks and gene modules, can be interactively queried through our web tool [19]; the genes and modules identified provide a basis for future investigation.

## Results

The results we report here are based on application of the GNAT algorithm to 1,559 samples from 35 tissues in the GTEx dataset. In each sample, we analyzed expression levels for 9,998 genes (Methods; S1 Table).

### Algorithm

The goal of our algorithm was to construct co-expression networks that captured both tissue-dependent and tissue-shared relationships between genes. In order to increase statistical power and accuracy when inferring such relationships in tissues with limited sample sizes, it used a two-stage transfer learning framework to construct networks for all tissues simultaneously. The first stage of the algorithm constructed a hierarchy over the tissues. The second stage optimized the network for each tissue using a method that encouraged fidelity to the expression data, sparsity in the networks, and similarity between networks that were nearby in the hierarchy.

**Fig 1:**
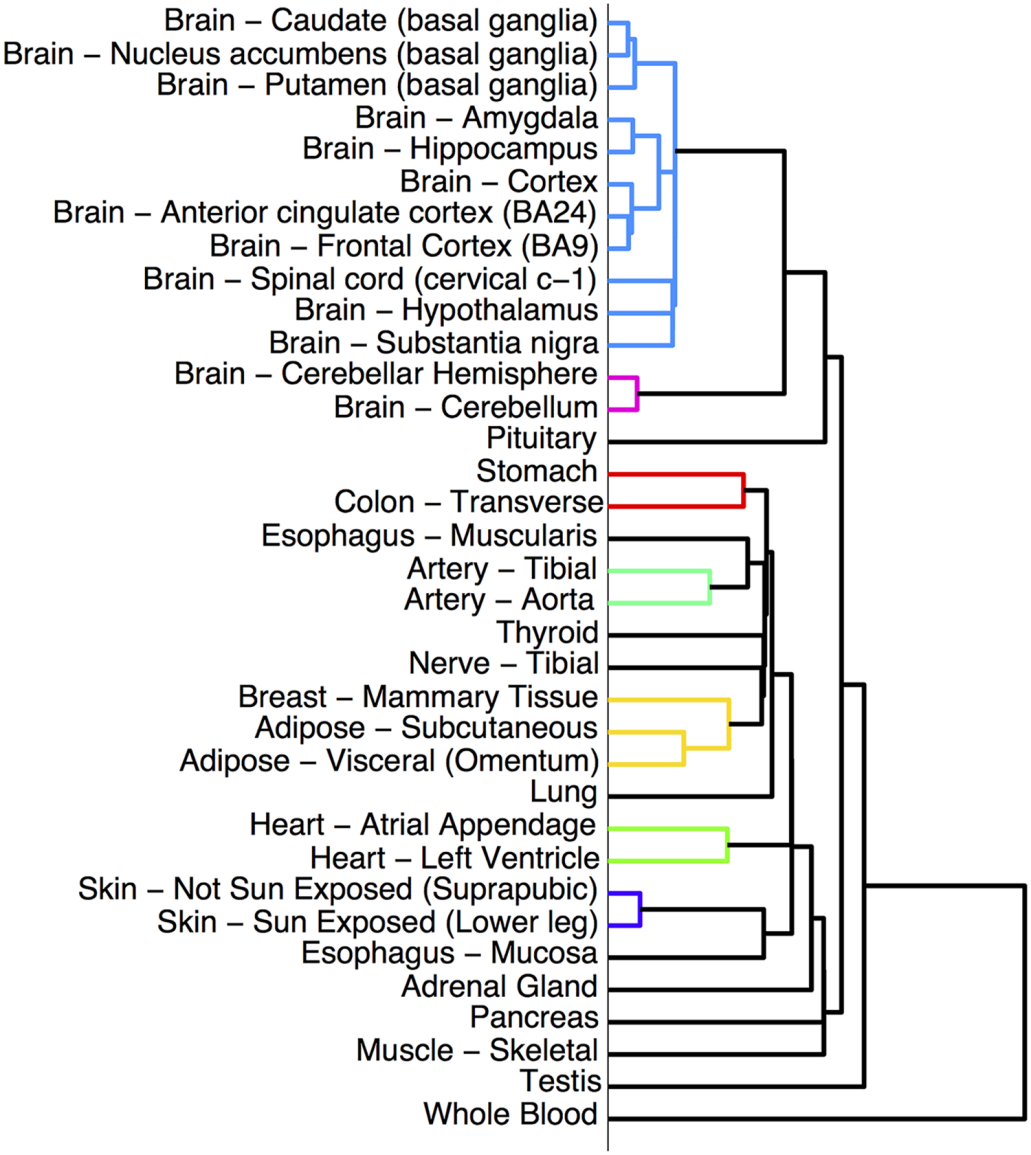
The hierarchy of tissues which is used as the basis for learning networks for each tissue. The hierarchy was created using hierarchical clustering: for each tissue, the mean expression of each gene in the tissue was computed, and tissues with similar gene expression patterns were merged into clusters. Lower branching points represent clusters with more similar gene expression patterns. Many biologically plausible clusters are apparent: the brain and non-brain cluster, and clusters for the basal ganglia, cortex, adipose tissue, heart, artery, and skin.

**1. Learning a hierarchy.** A tissue hierarchy was constructed using agglomerative hierarchical clustering on the mean gene expression levels for the 35 tissues (Fig 1). Since the rest of the algorithm was independent of the construction of the hierarchy, the method would also work with a hierarchy based on prior knowledge or on some other measure of dataset similarity.

**2. Learning networks based on the hierarchy.** We modeled the network for each tissue in the hierarchy using a Gaussian Markov Random Field (GMRF), a standard model in computational biology and image processing [20–22]. GMRFs model gene expression with a multivariate Gaussian distribution; we projected the samples for each gene onto a Gaussian (Methods) so this modeling assumption was reasonable. GMRFs are parameterized by an inverse covariance matrix *S* ^(*k*)^ (where *k* denotes the kth tissue) whose zero entries indicate pairs of genes that have expression levels which are conditionally independent given the expression levels of the other genes. These entries correspond exactly to direct connections between genes in the GMRF; other genes may still be connected through longer paths in the network. To encourage zero entries and diminish the number of links in the network, GMRFs maximize the convex Gaussian log likelihood plus an L1 sparsity penalty:

$$\begin{array}{c} \frac{n^{\left(k\right)}}{2}(\log\det S^{\left(k\right)}-tr\left(S^{\left(k\right)}\Sigma^{\left(k\right)}\right))-\lambda_{s}^{\left(k\right)}{\|S^{\left(k\right)}\|}_{1} \end{array}$$

where *n* ^(*k*)^ is the number of samples and Σ^(*k*)^ the empirical covariance matrix for the genes in tissue *k*, and $\lambda_{s}^{\left(k\right)}$ is a sparsity parameter. The sparsity makes the networks more interpretable and computationally tractable.

**Fig 2:**
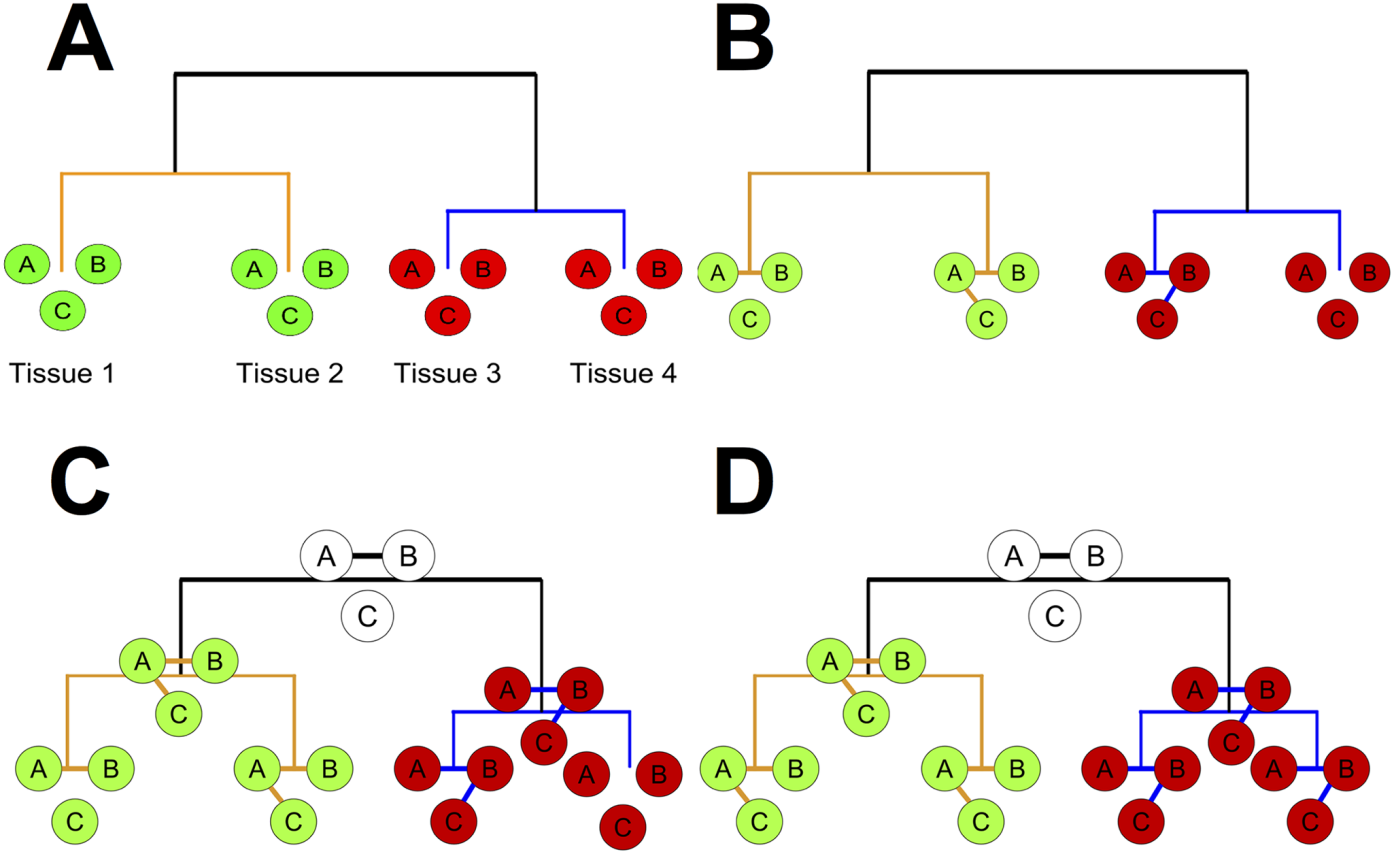
An illustration of our algorithm for hypothetical tissues (1, 2, 3, 4) and genes (A, B, C). The tree represents the hierarchy over tissues 1–4. For each tissue and each internal node in the hierarchy, gene networks over three genes (A, B, and C) are represented by circles (genes) and edges. a) Learning the hierarchy: tissues 1 and 2 are clustered together because A, B, and C have high mean expression levels in both tissues (green) and low levels in tissues 3 and 4 (red). b) co-expression networks are learned in each tissue independently. Edge AB is shared across three tissues; BC and AC only appear in one tissue. c) Networks are learned for each internal node in the hierarchy, representing an “average” of the child node networks, allowing similar tissues to share knowledge. The child node networks are re-learned and encouraged to be similar to their parents; this repeats until convergence. d) The final networks. Edge AB is now present in all 4 tissues; similarly, AC now appears in tissues 1 and 2, and edge BC in tissues 3 and 4.

We extended this method by constraining the matrices *S* ^(*k*)^ in tissues that were nearby in the hierarchy to have similar entries, creating similar networks, using an L2 penalty that penalized differences between the *S* ^(*k*)^. We used an L2 penalty rather than an L1 penalty because it allowed us to develop a fast parallel algorithm for optimizing the objective function (Methods). This transfer learning framework proved especially valuable for tissues with very few samples, for which we would otherwise lacked sufficient statistical power to infer co-expression networks. For example, we had only about two dozen samples for each of the 13 brain tissues in the GTEx dataset—too few to learn networks with 50 million parameters—but because all the brain tissues were closely related in our hierarchy, by adaptively sharing samples for related brain tissues we were able to make more robust estimates of co-expression. We provide a schematic illustration of our algorithm in Fig 2.

Previous work suggests the promise of using transfer learning to learn multiple genetic networks [18, 20, 21, 23]; hierarchical models have also been used more broadly throughout biology, for example to study phylogenies [24]. [18] used prior knowledge of a hierarchy of cancer cell types to learn a network for each cell type. Their method, however, relied on a hand-specified hierarchy, which would only be feasible if the number of datasets was smaller than the 35 in the GTEx dataset, and though successful in simulation was never shown to improve on prior methods on real data. [20] and [21] learn networks for multiple datasets using shrinkage between precision matrices, although they do not use a hierarchy and simply use a single shrinkage parameter. Additionally, none of these methods were designed to work on the large number of tissues included in the GTEx dataset, because such data has not been previously available. Importantly, our choice of optimization objective allows parallel optimization of all 35 tissue networks, which is critical for scaling to a large number of tissues. In contrast, the methods described in [18] and [20] cannot be easily parallelized and thus will not scale to the GTEx dataset, as we confirmed by testing their code on simulations with 35 tissues but far fewer genes than we use in our analysis (*n* = 10 versus *n* = 9998). Adapting our algorithm to the scale of the GTEx data required several further methodological innovations (Methods). For example, selecting a sparsity parameter for each of the 35 datasets using cross validation would have been prohibitively slow, so we developed a faster heuristic.

### Validation of Algorithm

**Fig 3:**
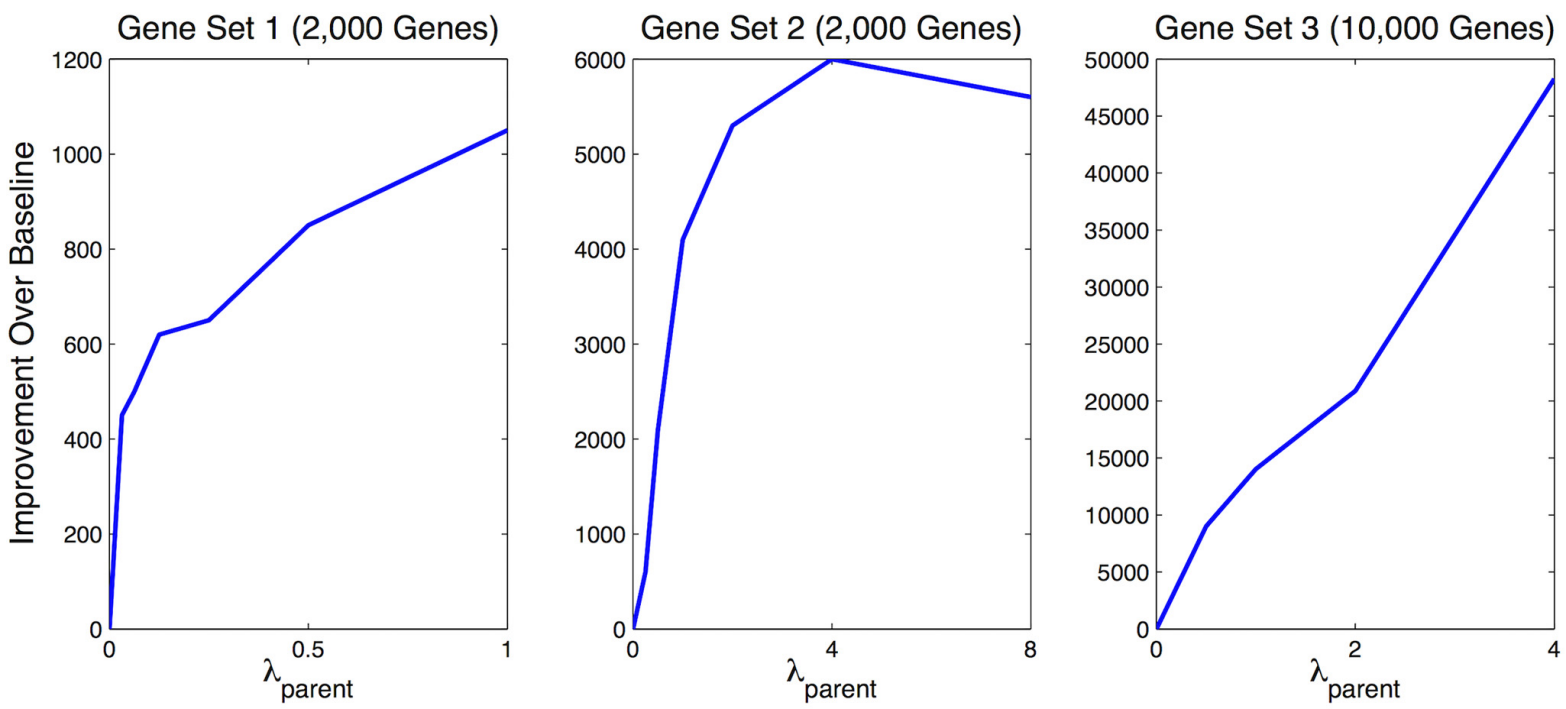
Network accuracy as measured by 5-fold cross validation. Learning networks independently corresponds to setting λ_*p*_ = 0 (the bottom left corner of each graph); the y-axis is the improvement in log likelihood over baseline. Our method improved on this baseline for all three gene sets we experimented with. The baseline of learning a single network for all tissues cannot be shown on this graph because its log likelihood is so low; we dropped it from further consideration in our analysis. The differing scales on the y-axes are due to the different sizes of the gene sets.

We used 5-fold cross-validation to evaluate our algorithm: for each tissue, we randomly divided our samples into five groups, learned networks based on samples from four of the five groups, and measured the accuracy of each network (quantified by the log likelihood on the held out test data) using the remaining group. We compared the performance of our method to two baselines: learning a network for each tissue independently, or learning a single network for all tissues. We observed a higher log likelihood on the held out test set using our approach as compared to the two baselines on three different gene sets of increasing sizes (Fig 3), indicating that the transfer learning approach resulted in a more robust estimation of the networks.

### Accuracy of Networks

We confirmed the accuracy of our learned networks in two ways. First, we evaluated agreement with two previous datasets. When we compared our networks to the co-expression database COEXPRESdb [25], pairs of genes we predicted to be linked had expression levels that were 2.6 times as correlated as genes we did not predict to be linked (*p* < 10^−6^, 2-sample KS test). To analyze tissue-specificity, we also compared our networks to TS-CoExp [12], which provides lists of tissue-specific co-expressed genes. Genes we predicted to be linked in a tissue were 10.5 times more likely to be linked in the corresponding TS-CoExp tissue than genes we did not predict to be linked (*p* < 10^−6^, *χ* ^2^ test). Links in the TS-CoExp database that were specific to a tissue were 2.1 times more likely to appear in our networks for the tissue than links in the TS-CoExp database that were not specific to that tissue (*p* < 10^−6^, *χ* ^2^ test). (We compared all these numbers to the baseline of the learning the networks independently, which yielded slightly higher agreement with TS-CoExp and virtually equivalent agreement with COEXPRESdb. We speculate that the higher agreement with TS-CoExp is due to the fact that the TS-CoExp networks were also learned on tissues independently.)

Second, using Gene Ontology [26], we found that genes that were linked in our networks were likely to represent functionally coherent interactions: across all tissues, genes that shared a Gene Ontology function were linked to each other 94% more often than were genes that did not share a function (*p* < 10^−6^, t-test). (Gene Ontology annotations were downloaded January 2012; for enrichment analysis, we only considered functional categories with 30–300 annotations.)

### Genes Important to Tissue Identity

**Fig 4:**
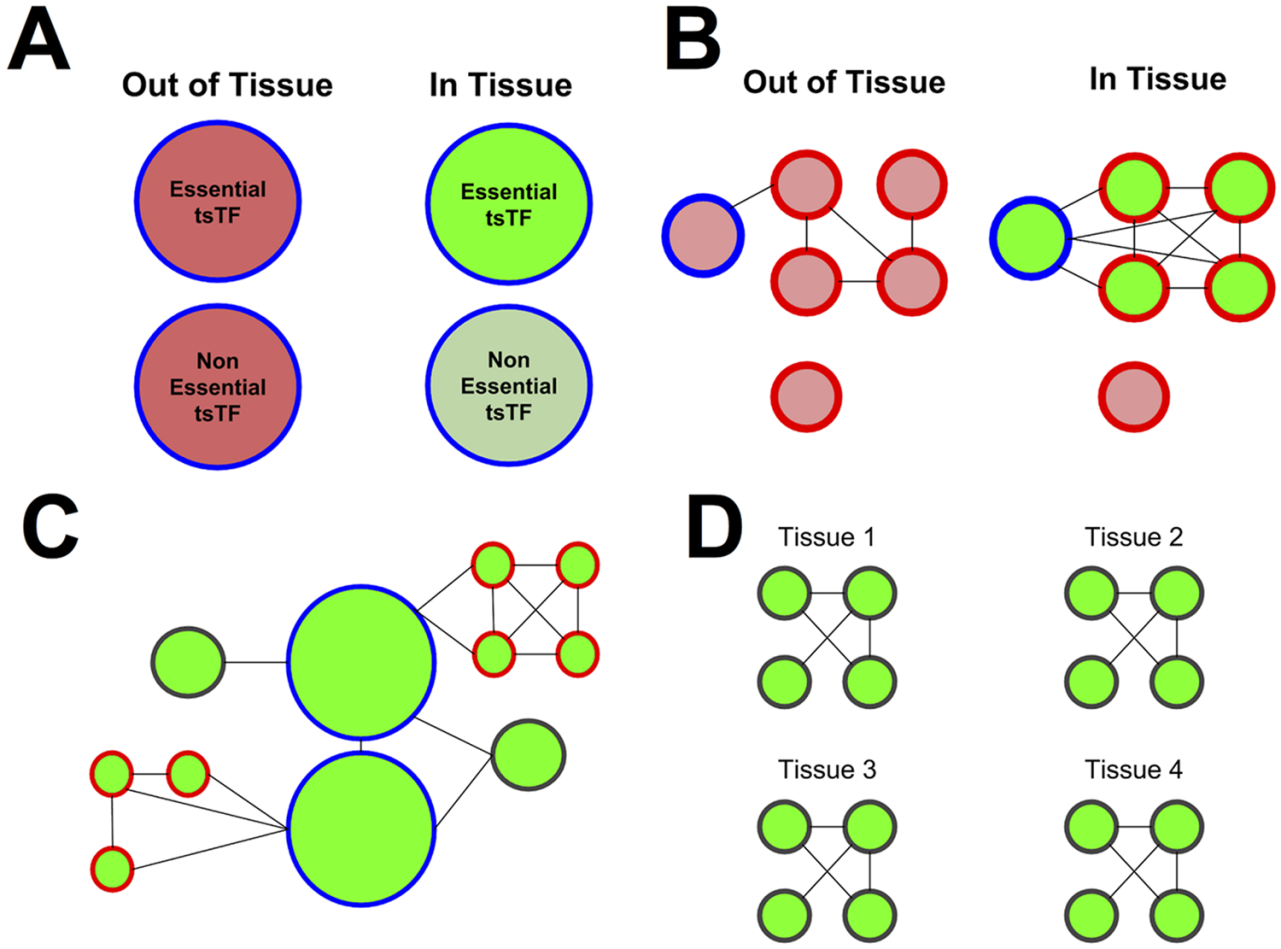
Important principles of tissue-specificity. a) Tissue-specific transcription factors (circled in blue) have higher expression levels (green) in tissues they are specific to, and those that change most dramatically in expression are most likely to be essential genes. b) Tissue-specific transcription factors connect to and upregulate genes with tissue-specific function (circled in red), which in turn connect to each other. c) Transcription factors lie at the centers of networks; genes with tissue-specific function and enriched modules lie at the network peripheries. d) Modules shared across tissues are more likely to be enriched for Gene Ontology functions, and tend to have functions common to all tissues like cell division.

Tissue-specific transcription factors (tsTFs) are important in defining tissue-specific phenotypes and mutations affecting tsTFs are enriched in loci associated with disease [2, 27]. We used our networks to analyze the role tsTFs play in tissue specificity using a collection of 203 known tsTFs (S2 Table) and 88 general TFs (gTFs) defined in [8]. We provide a schematic illustration of important conclusions of our analysis in Fig 4 and a tabular summary in Table 1.

**Table 1:** Summary of principles of tissue specificity.

Property	**Tissue-Specific Transcription Factors**	**General Transcription Factors**	**Genes with Tissue-Specific Functions**
**Higher-expressed than average gene?**	Yes (*p* < .001, 25/25 tissues)	No	Yes (*p* < .001, 27/29 tissues)
**Hubbier than average gene?**	Yes (*p* = .023, 20/25 tissues)	Yes (*p* < .001, 31/35 tissues)	Less hubby (*p* < .001, 23/29 tissues)
**Higher-expressed in tissues they’re specific to?**	Yes (*p* < .001, 10/10 gene sets)	NA	Yes (*p* < .001, 13/13 gene sets)
**Hubbier in tissues they’re specific to?**	No	NA	No

Changes of expression and hubness for transcription factors and genes with tissue-specific functions. All reported results were statistically significant by both a parametric (T) test and a non-parametric (bootstrap) test. As an additional confirmation, because gene sets in different tissues may have different properties, we also examined each gene set individually. We include the bootstrap probabilities in parentheses below, along with the proportion of gene sets/tissues for which the conclusion held true. To conduct the bootstrap comparisons, we compared values of expression and hubness for tsTFs, gTFs, and tsFXNGs to those for randomly selected set of genes in each tissue and repeated for 1000 iterates.

Well-connected genes (also known as “hubs”) are especially likely to be essential genes [28]. To quantify a measure of “hubness”, we computed the betweenness centrality [29] in our networks for each gene. Both general and tissue-specific TFs had higher average hubness scores than the average gene (*p* < .001, *p* = .023, respectively), highlighting the importance of TFs in our networks.

tsTFs were higher expressed in tissues they were specific to (*p* < .001, bootstrap; S1 Fig), and tsTFs that showed the largest expression increases in tissues they were specific to were especially likely to be essential genes as defined in [30] (16 of the top 20 tsTFs as compared to 115/203 tsTFs overall, *p* = .005, Fisher’s exact test; this enrichment was not sensitive to the choice of 20 as the cutoff). tsTFs which showed tissue-specific increases in expression tended to also show increases in hubness (Spearman *p* = 3 ⋅ 10^−4^) (S2 Fig).

To investigate how tsTFs interacted with genes with tissue-specific functions, we defined thirteen sets of tissue-specific function genes (tsFXNGs) using Gene Ontology annotations of gene function (S3 Table). Importantly, in our networks, tsTFs showed clear signs of preferentially connecting to and upregulating genes with tissue-specific functions. Across all tissues, tsTFs were 58% more likely to be linked to genes with tissue-specific functions than they were to be linked to other genes (*p* < 10^−6^, binomial test). Genes with tissue-specific functions that were connected to tsTFs were higher expressed on average than either a) genes with tissue-specific functions that were not connected to tsTFs or b) genes with non tissue-specific functions that were connected to tsTFs (*p* < 10^−6^, t-test). (For a list of the tsTFs linked to the largest numbers of tissue-specific genes, see S4 and S5 Tables). This underscores the important role that tfTFs play in upregulating genes with tissue-specific functions. Perhaps as a consequence of this upregulation, tsFXNGs were higher expressed in the tissues they were specific to than in the tissues they were not specific to (*p* < .001, bootstrap). (We note that because our analysis is correlative and our networks are undirected, further analysis is needed to conclusively establish directed regulatory relationships.)

Strikingly, in contrast to tsTFs, tsFXNGs were *less* hubby than the average gene. This was especially surprising given that, across all tissues, higher-expressed genes tended to be *more* hubby (*p* < .001, linear regression). However, our finding is consistent with prior research showing that tissue-specific proteins have fewer interactions than widely expressed proteins [31]. One possible explanation is that tsFXNGs lie at the periphery of our networks because they have specialized functions, acting as final nodes in pathways.

To gain further insights into genes that were important to tissue specificity, at each internal node in our tissue hierarchy (representing a point where one group of tissues split into two) we examined genes that differed in hubness most dramatically between the two tissue groups.

We first sorted all genes by the difference in their hubness in brain and non-brain tissues. The highest three scoring genes have all been previously shown to play important roles in the brain: ACTL6A, a chromatin remodeling factor which is required for the development of neural progenitors [32, 33]; VRK2, a gene implicated in schizophrenia [34]; and the Huntington’s gene, HTT. Notably, three of the four genes HTT was most often linked to in brain tissues are themselves associated with neurological disorders: RNF123 to major depression [35], MTHFR to neural tube defects [36] and dementia [37]; MECP2 to Rett syndrome [38]. HTT has been found to interact directly with MECP2 [39].

Several other tissue-specific hubs proved interesting (S6 Table). For example, the genes which increased most in hubness in the two skin tissues were APOE, which has been linked with skin lesions known as xanthomas [40] (although it is more famous because of its link with Alzheimer’s) and CERS3 [41], which when mutated causes congenital ichthyosis, a skin disease. Similarly, in the testis, the top-two ranked tissue-specific hubs were DDX3Y and KDM5D, both Y-chromosome linked genes which function in spermatogenesis [42–44].

### Modules Important to Tissue Identity

To identify tissue-specific and tissue-shared gene modules, we used the affinity propagation algorithm [45] to group genes into modules for each of the tissue networks. The average number of genes per module was 18, with the largest module containing 56 genes; there were 548 modules per tissue on average. 1,789 modules were enriched for Gene Ontology functions (Fisher’s exact test with Bonferroni correction *p* < .05); all enriched modules can be viewed online [19].

**Fig 5:**
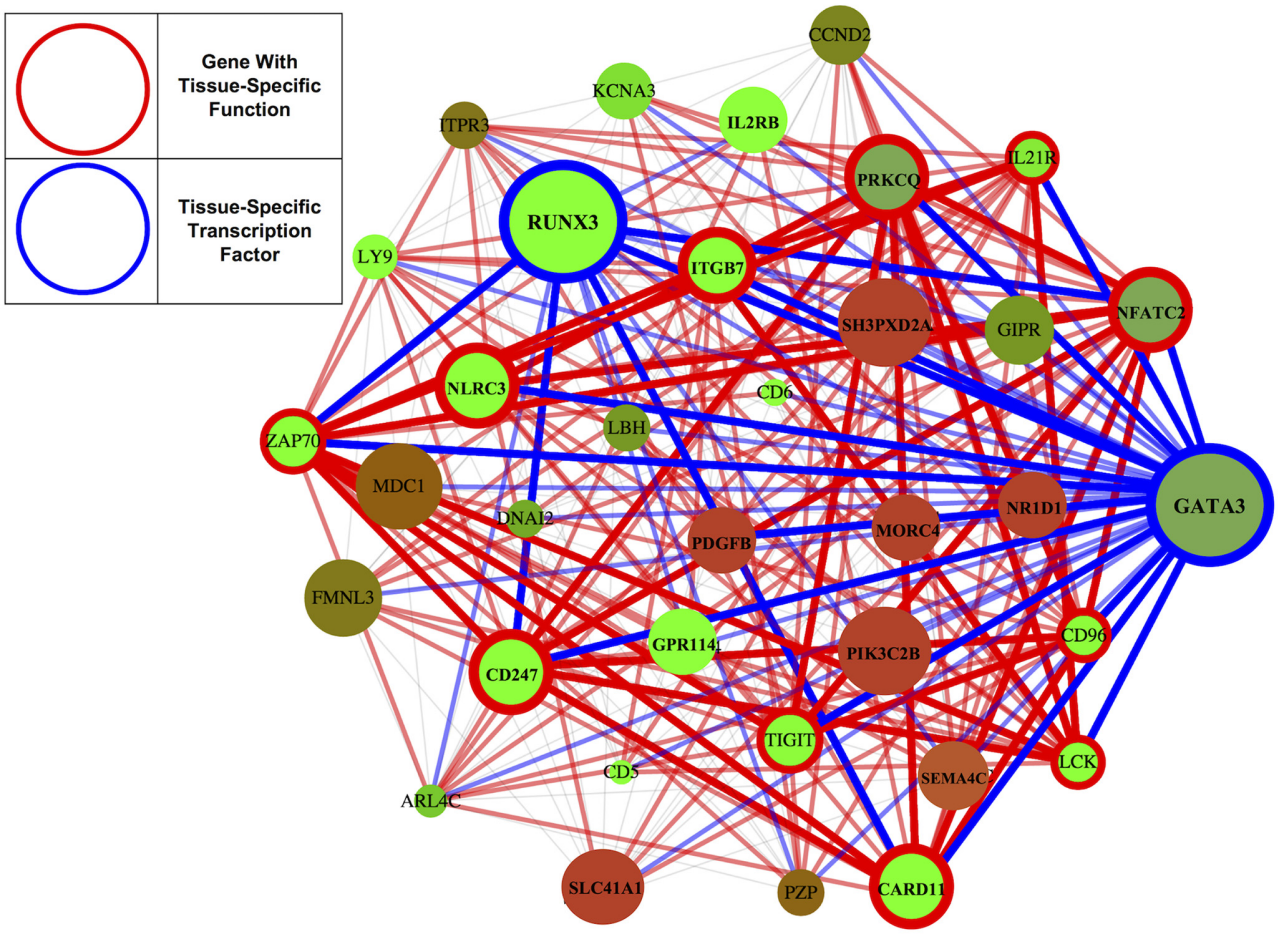
Genes linked to the blood-specific transcription factor GATA3 are enriched for immune function. Blue circles (and links) denote tsTFs; red circles denote tsFXNGs; the color of a gene indicates its level of expression, with green denoting upregulation and red denoting downregulation. This tightly connected cluster of genes comprises the blood-specific TFs GATA3 and RUNX3 (circled in blue) and 11 genes with immune related function (circled in red). GATA3 has been previously linked to RUNX3 [53] and implicated as a master regulator of the immune system [54], required for the maintenance of T cells; consistent with this, the set of genes linked to GATA3 to is significantly enriched for the T cell receptor signaling pathway and the T cell receptor complex (Fisher’s exact test with Bonferroni correction *p* = .0001 and .01, respectively) with 8 of the top 10 most enriched functions for these genes relating to the immune system.

Functionally enriched modules upregulated in a given tissue were often instrumental to tissue-specific function (S7 Table). In the blood, for example, the most upregulated enriched module (henceforth, the “top module”) was enriched for T cell receptor complex expression (Fig 5); in the skin, for epidermis development; in the testis, for chromosome segregation; in the muscle and heart for muscle-related functions; and in various brain tissues for glutamate receptor activity, chloride channel activity, and regulation of axonogenesis. Given the plausibility of these functions, these modules represent useful candidates for future investigation.

Curiously, genes that were members of enriched clusters were less hubby than genes that were not in every tissue (*p* < .001, t-test). This discrepancy was so pronounced that we originally noticed it by visual examination of the networks in our web tool. One explanation would be that these enriched modules, like tsFXNGs, lie at the peripheries of networks because they act as the final steps in functional pathways.

Top modules also revealed more complex relationships between tissues. For example, immune-related modules were found not only in the blood, but also in lung and digestive tissues. (We note that there is some possibility of sample contamination, with the collected lung tissue including some blood cells. On the other hand, previous research [5] has found that the lung has similar gene expression patterns to immune tissues like the spleen and thymus, perhaps indicating the importance of immune function in the lung.) The top module in suprapubic skin, enriched for mitosis, was also upregulated in other tissues where cells divide frequently, including the testis, the stomach, the esophagus, and the colon.

**Fig 6:**
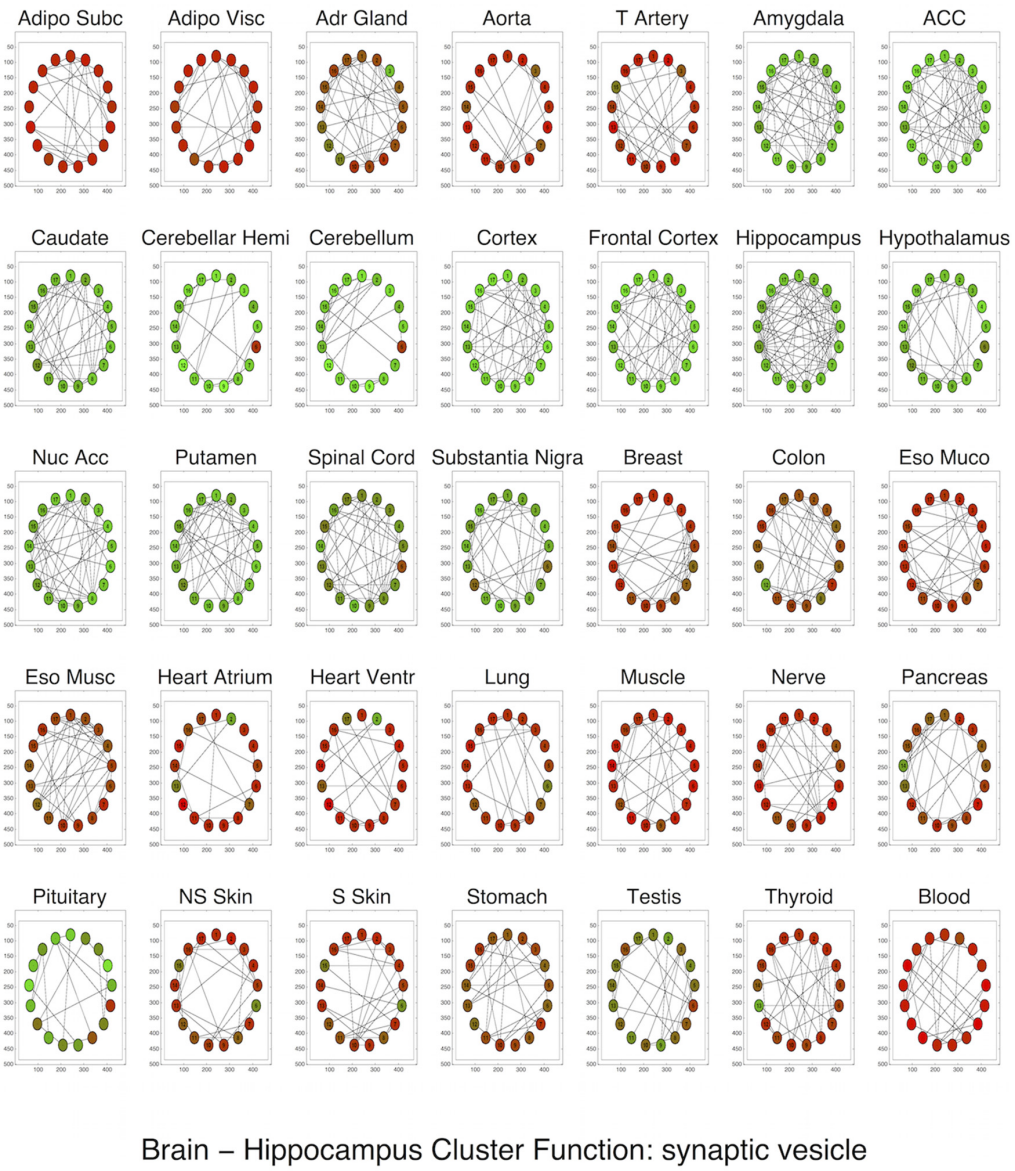
The most upregulated enriched cluster in the hippocampus, enriched for synaptic vesicle function, shown across all tissues. Green indicates upregulation of a gene: the cluster is upregulated in all brain tissues (including the pituitary) and downregulated in non-brain tissues.

Our analysis also revealed upregulation of tissue-specific modules in “similar” tissues: the top module in one tissue was often upregulated in tissues nearby in the hierarchy. For all brain tissues, top modules were dramatically upregulated in all other brain tissues as well, but not in non-brain tissues (Fig 6). The top module in the heart atrium, related to “structural constituent of muscle” was unsurprisingly upregulated in the muscle and heart ventricle as well.

We also identified a number of modules that were conserved in most tissues, representing ubiquitous functions shared by all cells. For each module in each tissue, we measured the degree to which the module was conserved by calculating the average fraction of links that were present among its genes in other tissues: $f=\frac{1}{K}\sum_{j=1}^{K}\frac{n_{k}}{n}$, where *K* was the total number of tissues, *n*_*k*_ was the number of links between genes in the module in the kth tissue, and *n* was the number of links had the module been fully connected. When we sorted modules by *f* (filtering out modules with fewer than 10 genes, which tended to have high interlink fractions) we found that the top 50 modules were much more likely than the average module to be significantly enriched for a Gene Ontology function (78% vs 11%), and were dominated by functions related to chromosome segregation or the cell cycle, capacities essential for almost every tissue. When we sorted functions by the degree to which their enriched modules were conserved, we found that 8 of the 10 most conserved functions were general to almost every tissue, relating to cell division or cell signaling: “phosphatidylinositol-mediated signaling”, “mitotic cell cycle spindle assembly checkpoint”, “chromosome segregation”, “cell cycle”, “transport”, “cytokinesis”, “M phase of mitotic cell cycle”, and “chromosome, centromeric region”.

## Discussion

We present an algorithm that infers genetic networks in a collection of tissues, using a hierarchy to share data between tissues with many samples and tissues with few, and show that this sharing increases the accuracy with which we infer the networks. We use an objective function that can be optimized over all tissues in parallel, allowing our algorithm to scale to the GTEx dataset, and propose several further innovations that increase scalability. Our algorithm has broad applicability to any dataset of hierarchically related samples: species in a phylogenetic tree or cell lineages in a tumor, for example.

We then conduct a detailed analysis of the genetic networks in 35 human tissues, searching for principles underlying both the unity and diversity of tissue function. We find that unity arises from modules that persist across tissues, which are not only disproportionately likely to be enriched for Gene Ontology functions, but for functions like mitosis that are shared across virtually every tissue. We show that previously discovered general transcription factors, which act across many tissues, tend to be hubs in our networks.

At the same time, we find strong evidence of functional specialization among tissues (Fig 4). tsTFs, which tend to be hubs in our networks, play instrumental roles: they preferentially connect to genes with tissue-specific functions, and these genes show higher expression levels. Strikingly, genes with tissue-specific functions lie at the peripheries of our networks, as do genes within enriched clusters; one explanation for this is that these genes act as the final steps in pathways instrumental to tissue-specific function. Finally, modules enriched for Gene Ontology functions that are upregulated within a tissue are often instrumental to tissue-specific function, and provide intriguing candidates for biological investigation. As the availability of biological data increases, statistical network analysis will continue to reveal both important general principles by which networks accomplish their functions, and specific hypotheses worth investigating.

## Materials and Methods

### GTEx Data

Genome-wide gene expression data for 1,606 samples across 43 unique tissues was collected by the GTEx consortium using RNA-sequencing; we used version phs000424.v3.p1 of the data. We confined our analysis to tissues with expression data for at least ten samples, resulting in a total of 1,559 samples and 35 tissues (S1 Table).

### Gene Ontology Data

GO annotations were downloaded from www.geneontology.org on January 28th, 2012. All IEA annotations were excluded, and then all remaining GO categories with 20–300 annotated genes (any annotation type except IEA) were included in the analysis. No filter was placed on the ontology.

### Normalization of Data

For each read count *n*_*i*_ in each sample, we computed the normalized read count *r*_*i*_ = *log*_2_(2 + *C* ⋅ *n*_*i*_/*n*) where *n* was the total number of reads in the sample and *C* was the FPKM normalization constant, 5 ⋅ 10^7^. Because GMRFs are designed for Gaussian data, we projected all samples for each transcript for each tissue onto a Gaussian with variance 1.

### Selection of Gene Set

The GTEx dataset contained expression levels for 52,576 different transcripts, which would have produced a prohibitively large covariance matrix. We filtered down the set of transcripts to a more computationally tractable size. Since transcripts would have to show variation in expression levels to have meaningful patterns in correlation, we first filtered out all probes that were zero or constant across any tissue by requiring that genes show non-zero expression in at least 1/5 of samples in a tissue. We then selected a set of transcripts as follows: we repeatedly looped over all tissues, and for each tissue selected the transcript which corresponded to a gene which showed the highest relative expression in that tissue, was annotated in Gene Ontology, and was not already included in the genes selected. (We defined relative expression in a tissue to be the difference between the gene’s mean expression in that tissue and the gene’s mean expression across all tissues divided by the variance of the gene’s expression). We continued this process until we had obtained 9,998 genes. (This number was produced by choosing a threshold of 10,000 genes, which represented a compromise between representing the entire dataset and achieving computational tractability, and removing two genes which did not have unique names.) This process yielded a set of genes with diverse tissue-specific functions (since each tissue contributed many genes which showed high relative expression in that tissue).

We confirmed that our algorithm also produced improvements over the baseline algorithms in two smaller gene sets containing roughly 2,000 genes: one selected using the method described above, and one selected using the genes that showed the largest variance across tissues.

### Optimization of Networks Given Hierarchy

Given a hierarchy of *K* tissues, our algorithm learned a precision matrix for each node in the hierarchy, including the *K* leaf nodes *S* ^(1)^, *S* ^(2)^, …, *S* ^(*K*)^ (which corresponded directly to tissues) and the *K* − 1 internal nodes *S* ^(*K*+1)^, …, *S* ^(2*K*−1)^. Denote by $S_{p}^{k}$ the parent of node *k*. Then the optimization objective was

$$\begin{array}{c} \max_{S^{\left(k\right)},k=1,\ldots ,2K-1}\sum_{k=1}^{K}(\frac{n^{\left(k\right)}}{2}(\log\det S^{\left(k\right)}-tr\left(S^{\left(k\right)}\Sigma^{\left(k\right)}\right))-\lambda_{s}^{\left(k\right)}{\|S^{\left(k\right)}\|}_{1})-\lambda_{p}\sum_{k=1}^{2K-2}{\|S^{\left(k\right)}-S_{p}^{\left(k\right)}\|}_{2}^{2} \end{array}$$

$$\begin{array}{c} S^{\left(k\right)}⪰0,k=1,2,\ldots ,K \end{array}$$

where $\lambda_{s}^{\left(k\right)}$ were the *k* L1 sparsity penalties (chosen for each dataset as described below) and λ_*p*_ was the L2 penalty that encouraged *S* ^(*k*)^ to be similar to its parent $S_{p}^{\left(k\right)}$ (constant for all tissues). In other words, for the leaf nodes, our optimization objective included the Gaussian log likelihood term, a sparsity penalty on the off-diagonal elements, and an L2 parent similarity term; for the internal nodes, there was only an L2 similarity term. While this optimization objective was convex, the inverse precision matrices had tens of millions of entries and optimizing all 2*K* − 1 matrices simultaneously would have been very slow. Instead, we used an iterative algorithm: given a hierarchy, the full optimization procedure was as follows:

1. For each dataset *k* = 1, …, *K*, learn an initial *S* ^(*k*)^ by maximizing
$\frac{n^{\left(k\right)}}{2}\left(\text{log det}S^{\left(k\right)}-tr(S^{\left(k\right)}\Sigma^{\left(k\right)})\right)-\lambda_{s}^{\left(k\right)}{\left\|S^{\left(k\right)}\right\|}_{1}$. In other words, initialize by solving the graphical lasso problem for each dataset independently.
2. Until convergence:
  - Optimize the internal matrices, *S* ^(*k*)^, *k* = *K* + 1, …, 2*K* − 1, holding the leaf matrices fixed; because all relevant terms of the objective were quadratic, this was analytic and essentially instantaneous. (We note that this would not be true if an L1 penalty were used rather than an L2 penalty.)
  - Optimize the leaf matrices, *S* ^(*k*)^, *k* = 1, …, *K*, holding the internal matrices fixed; each leaf matrix was independent of the others given its parent, so this was done in parallel. Optimization was performed using the L1General [46] and glasso [47] packages.

To ensure that the size of the entries in *S* were comparable across tissues and between internal and external nodes, prior to each iteration we normalized each *S* such that all *S* had the same mean absolute value of diagonal elements and the same mean absolute value of nonzero off-diagonal elements.

### Sparsity Parameter Selection

To expedite this potentially lengthy process of choosing a sparsity parameter $\lambda_{s}^{\left(k\right)}$ for each of 35 tissues, we used a heuristic rather than using the traditional cross-validation for every single tissue. We confirmed that our heuristic produced similar results to cross validation. [48] found the BIC penalty effective in selecting the sparsity parameter for graphical lasso: *log*(*n*)‖*S* ^(*k*)^‖_0_, where ‖*S* ^(*k*)^‖_0_ is the number of non-zero off-diagonal entries of *S* ^(*k*)^. This suggests setting $\lambda_{s}^{\left(k\right)}$ to a value that makes the L1 penalty equal to the BIC penalty: $\lambda_{s}^{\left(k\right)}=log(n^{\left(k\right)})/\bar{s}$, where $\bar{s}$ is the mean absolute value of the nonzero off-diagonal entries in the optimized precision matrix. Substantiating this, we found that *log*(*n* ^(*k*)^) was tightly correlated in both simulated and actual data with the optimal L1 penalty, and also outperformed the $\sqrt{n^{\left(k\right)}}$ suggested by [49]. This appears to beg the question of how to estimate $\bar{s}$ without doing the actual optimization; however, we found that $\bar{s}$ was tightly correlated in both simulations and in the GTEx datasets with $\bar{\Sigma }$, the mean size of the entries in the empirical covariance matrix. Similarly, $log(n^{\left(k\right)})/{\bar{\Sigma }}^{\left(k\right)}$ was tightly correlated in both simulations and actual data with $\lambda_{s}^{\left(k\right)}$. Thus, we can select $\lambda_{s}^{\left(k\right)}$ for all *K* datasets by using parameter search to select $\lambda_{s}^{\left(1\right)},\lambda_{s}^{\left(2\right)},\ldots \lambda_{s}^{\left(i\right)}$, where *i* is much smaller than *K*; we then do a regression of the optimized $\lambda_{s}^{\left(k\right)}$s on $log(n^{\left(k\right)})/{\bar{\Sigma }}^{\left(k\right)}$, and use that fit to compute the remaining $\lambda_{s}^{\left(k\right)}$. We confirm that this method works on both simulated precision matrices and the GTEx dataset. For the GTEx dataset, using *i* = 5 yields λ^(*k*)^ within 17% of the values selected by cross-validation on average; *i* = 3 yields values within 26%, acceptable discrepancies given the coarseness of parameter search.

### Constraining Precision Matrices to Be Block-Diagonal

Most algorithms for solving the graphical lasso problem with *p* genes are *O*(*p* ^3^), making optimization intractable for 9,998 genes. If the optimal solution were block diagonal, with block sizes *p*_1_, …, *p*_*k*_, optimization could be performed in $O\left(\sum_{i=1}^{k}p_{i}^{3}\right)$, as noted in [50] and [51]. Unfortunately, we found that the criterion these papers provide for determining whether the problem decomposes requires too large a sparsity parameter to be practically useful. Instead, we used an approximate eigenvector-based diagonalization similar to that described in [52]: for each tissue, we computed a matrix *C* ^(*k*)^, with $C_{ij}^{\left(k\right)}=max{\left(0,\Sigma_{ij}^{\left(k\right)}-\lambda_{s}^{\left(k\right)}\right)}^{2}$. We then computed the weighted sum of the matrices: $S=\sum_{k=1}^{K}n^{\left(k\right)}C^{\left(k\right)}$, and partitioned *S* into approximate connected components using the principal eigenvector as described in [52]. (To ensure that all components had tractable size, we set a maximum component size of 500 genes and recursively partitioned components until they fell below this threshold.) We confirmed that this approximate solution had a higher test log likelihood than that obtained by choosing a sparsity parameter sufficiently large to make an exact solution tractable.

### Robustness to Perturbation in the Algorithm

Because the L1 optimization algorithm and our initializations are stochastic, the final optimized networks may vary slightly from run to run. However, we verified that our results were not overly sensitive to repeated runs of the algorithm, to parameter settings, or to which samples we used by examining two modified networks: one optimized using a subset of 4/5 of the samples and one optimized using λ_*p*_ = 2 as opposed to λ_*p*_ = 4. We found that both modified networks were highly enriched for links in our actual network; links in the actual network were more than 100 times as likely as random links to be found in the modified networks. In modified networks, we tested a number of the network properties reported above. First, we verified that we still saw statistically significant correlations with the external datasets COEXPRESdb and TS-CoExp. Second, we verified that tissue-specific genes, and genes with shared functions, still showed statistically significant tendencies to be linked to each other. Finally, we verified that tsTFs still showed a statistically significant tendency to be linked to genes with tissue-specific functions. The robustness of all these conclusions made us confident that the conclusions reported above are unlikely to be due to which samples in the dataset are used, the values of the parameters, or variations in the initialization of the algorithm, although specific links in the networks may change.

We also analyzed the proportion of links that were conserved across different conditions. We compared networks calculated using our chosen value of λ_*p*_ = 4 to those learned with different values of λ_*p*_ (S9 Table); 89% of links were conserved between networks learned with λ_*p*_ = 4, λ_*p*_ = 2, and 98% between networks learned with λ_*p*_ = 4, λ_*p*_ = 8. A somewhat lower proportion (75%) of links were conserved between λ_*p*_ = 4, λ_*p*_ = 0, implying that the use of a similarity penalty may be more important than the exact size of the similarity penalty. We also compared the networks learned on all samples to the networks learned using a subset of 4/5 of the samples; 38% of the links were conserved in the average tissue. Given the sparsity of the networks, all these proportions are more than 100 times what random chance would predict. However, because specific links can change depending on which samples are used, the broad conclusions of our analysis are more robust than any particular link we predict.

## Supporting Information

S1 Fig Tissue-specific transcription factors are upregulated in tissues they are specific to. Each row is a tissue; each column is a tissue-specific transcription factor set; the color of a square denotes the mean expression of the transcription factor set in the tissue, with green denoting upregulation and red denoting downregulation.
(TIFF)

S2 Fig Tissue-specific transcription factors plotted by their increase in hubness and standardized gene expression in tissues they are specific to. Most tissue-specific transcription factors increase in expression in tissues they are specific to, and those that increase in expression also tend to increase in hubness. Transcription factors that are essential genes are marked in red; the “top” transcription factors that show the largest tissue-specific increases in expression are especially likely to be members of this essential gene set (16/20 top transcription factors as compared to 115/203 transcription factors overall). For clarity, only the top TFs are labeled.
(TIFF)

S1 Table The number of samples for each tissue in our dataset. (XLSX)

S2 Table Specific and general transcription factors used in analysis. Specific transcription factors have been found to be specific to one particular tissue or set of tissues; general transcription factors have been found to be active across many tissues.
(XLSX)

S3 Table Genes with tissue-specific functions, as identified by GO keyword. We defined 13 sets of tissue-specific genes. For all sets except for brain tissues, we defined keywords associated with tissue-specific functions (third column of table), selected all GO annotations that contained these keywords (some keywords produced no matches), and manually inspected all GO annotations to remove any false positive annotations. (Eg, “uterine wall breakdown” would be falsely associated with the stomach due to the “breakdown” keyword, but removed by manual curation.) We defined “tissue-specific function genes” as all genes that were associated with these tissue-specific GO annotations. Because of the importance of brain tissues to the analysis, we identified brain-specific genes by individually examining all 1614 GO functions and identifying 109 brain-related ones; the large number of brain genes is due to the fact that we selected genes which were highly expressed in our tissues. We provide the most common brain-related GO functions in the table above.
(XLSX)

S4 Table Tissue-specific transcription factors which were linked to an especially large number of genes of interest—tissue-specific genes, tissue-specific transcription factors, or general transcription factors. We filtered for transcription factors which were a) linked to at least three genes of interest and b) linked to at least twice as many genes of interest as random chance would predict. We include the unadjusted binomial probability that each transcription factor would be linked to at least that many genes of interest by chance.
(XLSX)

S5 Table Brain-specific transcription factors which are linked to an unusual number of genes of interest. For each transcription factor, we give the brain tissues in which this occurs as well as the brain tissue in which it is most linked to brain-specific genes and the unadjusted binomial probability in that tissue.
(XLSX)

S6 Table The genes which differ most dramatically at important split points in the hierarchy, as measured by changes in expression and hubness. (XLSX)

S7 Table The GO functions of the three most upregulated enriched clusters in each tissue. (XLSX)

S8 Table Tissue-specific GO annotations selected using keyword search and manual curation. (XLSX)

S9 Table The fraction of links preserved between networks learned with different values of λ_*p*_. The second column denotes the percentage of links that appear in the first network that also appear in the second; the third column denotes the ratio of observed shared links to shared links expected if there were no relationship between the networks.
(XLSX)

S1 Text This file provides the full list of authors and affiliations for the GTEx Consortium. (DOCX)

S1 Data This file provides a MATLAB implementation of the algorithm described in the paper. (ZIP)
