# Supplementary figures and images for: Sharing and Specificity of Co-expression Networks across 35 Human Tissues
Source: PLoS Comput Biol. 2015 May 13;11(5):e1004220. doi: 10.1371/journal.pcbi.1004220 (PMC4430528; doi:10.1371/journal.pcbi.1004220)

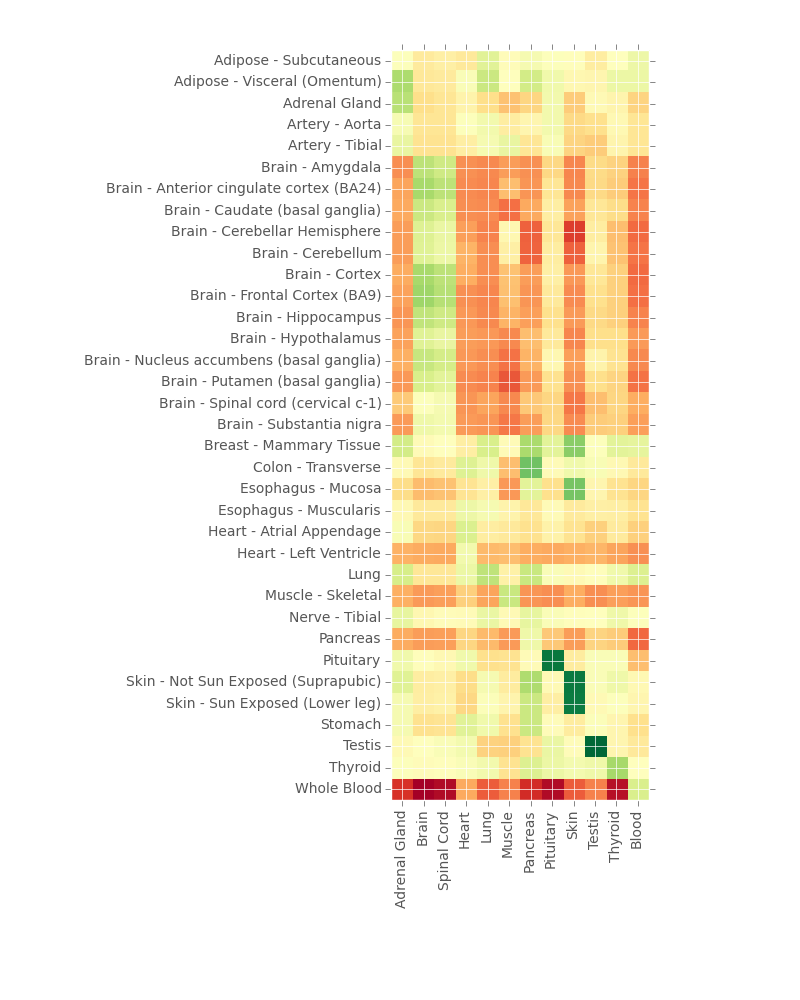

Supplement: S1 Fig — Each row is a tissue; each column is a tissue-specific transcription factor set; the color of a square denotes the mean expression of the transcription factor set in the tissue, with green denoting upregulation and red denoting downregulation. (TIFF) [file pcbi.1004220.s001.tiff]

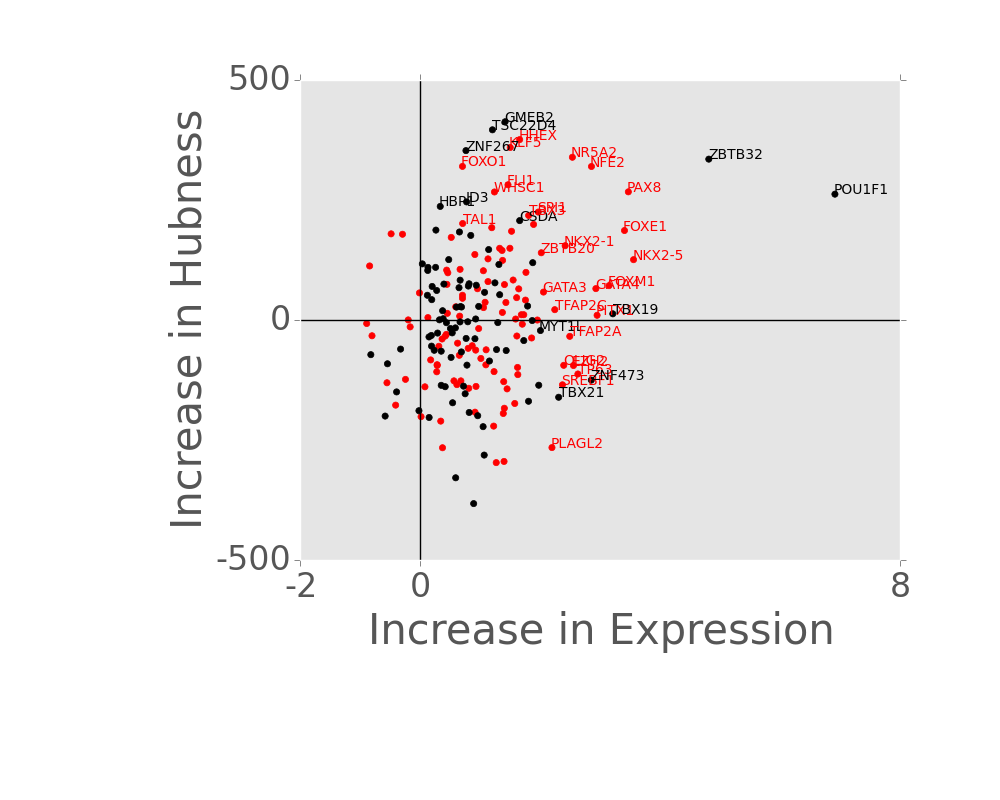

Supplement: S2 Fig — Most tissue-specific transcription factors increase in expression in tissues they are specific to, and those that increase in expression also tend to increase in hubness. Transcription factors that are essential genes are marked in red; the “top” transcription factors that show the largest tissue-specific increases in expression are especially likely to be members of this essential gene set (16/20 top transcription factors as compared to 115/203 transcription factors overall). For clarity, only the top TFs are labeled. (TIFF) [file pcbi.1004220.s002.tiff]
