# Supplementary material for: Sharing and Specificity of Co-expression Networks across 35 Human Tissues
Source: PLoS Comput Biol. 2015 May 13;11(5):e1004220. doi: 10.1371/journal.pcbi.1004220 (PMC4430528; doi:10.1371/journal.pcbi.1004220)
Supplement: S1 Text — (DOCX) [file pcbi.1004220.s012.docx]

**The GTEx Consortium**

(Arranged by area of contribution and then by institution)

**Analysis working group: LDACC** Kristin G. Ardlie^1^, David S. Deluca^1^, Ayellet V. Segrè^1^, Timothy J. Sullivan^1^, Taylor R. Young^1^, Ellen T. Gelfand^1^, Casandra A. Trowbridge^1^, Julian B. Maller^1,2^, Taru Tukiainen^1,2^, Monkol Lek^1,2^, Lucas D. Ward^1,3^, Pouya Kheradpour^1,3^, Benjamin Iriarte^3^, Yan Meng^1^, Cameron D. Palmer^1,4^, Wendy Winckler^1^, Joel Hirschhorn^1,4^, Manolis Kellis^1,3^, Daniel G. MacArthur^1,2^, Gad Getz^1,6^; **UNC/NCSU** Andrey A. Shablin^7^, Gen Li^8^, Yi-Hui Zhou^9^ Andrew B. Nobel^8^, Ivan Rusyn^10,11^, Fred A. Wright^9^; **U Geneva** Tuuli Lappalainen^12,13,14,15,16,17^, Pedro G. Ferreira^12,13,14^, Halit Ongen^12,13,14^, Manuel A. Rivas^18^, Alexis Battle^19,20^, Sara Mostafavi^19^, Jean Monlong^21,22,23^, Michael Sammeth^21,22,24^, Marta Mele^21,22,25^, Ferran Reverter^21,26^, Jakob Goldmann^21,27^, Daphne Koller^19^, Roderic Guigo^21,22,28^, Mark I. McCarthy^18,29,30^, Emmanouil T. Dermitzakis^12,13,14^; **U Chicago** Eric R. Gamazon^31^, Hae Kyung Im^31^, Anuar Konkashbaev^31^, Dan L. Nicolae^31^, Nancy J. Cox^31^; **U Chicago** Timothée Flutre^32,33^, Xiaoquan Wen^34^, Matthew Stephens^32,35^, Jonathan K. Pritchard^32,36,37^; **Harvard** Zhidong Tu^38,39^, Bin Zhang^38,39^, Tao Huang^38,39^, Quan Long^38,39^, Luan Lin^38,39^, Jialiang Yang^38,39^, Jun Zhu^38,39^, Jun Liu^40^.

**Biospecimen and data collection, processing, quality control, storage, and pathological review: caHUB Biospecimen Source Sites: NDRI** Amanda Brown^41^, Bernadette Mestichelli^41^, Denee Tidwell^41^, Edmund Lo^41^, Mike Salvatore^41^, Saboor Shad^41^, Jeffrey A. Thomas^41^, John T. Lonsdale^41^; **Roswell Park** Christopher Choi^42^, Ellen Karasik^42^, Kimberly Ramsey^42^, Michael T. Moser^42^, Barbara A. Foster^42^, Bryan M. Gillard^42^; **Science Care Inc.** John Syron^43^, Johnelle Fleming^43^, Harold Magazine^43^; **Gift of Life Donor Program** Rick Hasz^44^; **LifeNet Health** Gary D. Walters^45^; **UNYTS** Jason P. Bridge^46^, Mark Miklos^46^, Susan Sullivan^46^. **caHUB ELSI Study: VCU** Laura K. Barker^47^, Heather Traino^47^, Magboeba Mosavel^47^, Laura A. Siminoff^47,48^. **caHUB Comprehensive Biospecimen Resource: Van Andel** Dana R. Valley^49^, Daniel C. Rohrer^49^, Scott Jewel^49^. **caHUB Pathology Resource Center: NCI** Philip Branton^50,51^; **Leidos Biomedical Research Inc.** Leslie H. Sobin^52^. **caHUB Comprehensive Data Resource: Leidos Biomedical Research Inc**. Liqun Qi^52^, Pushpa Hariharan^52^, Shenpei Wu^52^, David Tabor^52^, Charles Shive^52^.

**caHUB Operations Management: Leidos Biomedical Research Inc.** Anna M. Smith^52^, Stephen A. Buia^52^, Anita H. Undale^52^, Karna L. Robinson^52^, Nancy Roche^52^, Kimberly M. Valentino^52^, Angela Britton^52^, Robin Burges^52^, Debra Bradbury^52^, Kenneth W. Hambright^52^, John Seleski^52^, Greg E. Korzeniewski^52^; **Sapient Government Services** Kenyon Erickson^53^.

**Brain Bank Operations: University of Miami** Yvonne Marcus^54^, Jorge Tejada^54^, Mehran Taherian^54^, Chunrong Lu^54^, Barnaby E. Robles^54^, Margaret Basile^54^, Deborah C. Mash^54^.

**Program Management: NHGRI** Simona Volpi^51,55^, Jeffery P. Struewing^51,55^, Gary F. Temple^51,55^, Joy Boyer^51,56^, Deborah Colantuoni^51,55^; **NIMH** Roger Little^51,57^, Susan Koester^51,58^; **NCI** Latarsha J. Carithers^51,59^, Helen M. Moore^51,59^, Ping Guan^50,51^, Carolyn Compton^51,59^, Sherilyn J. Sawyer^50,51^, Joanne P. Demchok^50,51^, Jimmie B. Vaught^50,51^, Chana A. Rabiner^50,51^, Nicole C. Lockhart^50,51^.

**Writing Committee:** Kristin G. Ardlie^1^, Gad Getz^1,6^, Fred A. Wright^9^, Manolis Kellis^1,3^, Simona Volpi^51,55^, Emmanouil T. Dermitzakis^12,13,14^.

^1^The Broad Institute of Massachusetts Institute of Technology and Harvard University, Cambridge, Massachusetts 02142, USA. ^2^Analytic and Translational Genetics Unit, Massachusetts General Hospital, Boston, Massachusetts 02114, USA. ^3^MIT Computer Science and Artificial Intelligence Laboratory, Massachusetts Institute of Technology, Cambridge, Massachusetts 02139, USA. ^4^Department of Genetics, Boston Children's Hospital, Boston, Massachusetts 02115, USA. ^6^Cancer Center and Department of Pathology, Massachusetts General Hospital, Boston, Massachusetts, 02114, USA. ^7^Center for Biomarker Research and Personalized Medicine, Virginia Commonwealth University, Richmond, Virginia 23298, USA. ^8^Department of Statistics and Operations Research and Department of Biostatistics, University of North Carolina, Chapel Hill, North Carolina 27599, USA. ^9^Bioinformatics Research Center and Departments of Statistics and Biological Sciences, North Carolina State University, Raleigh, North Carolina 27695, USA. ^10^Department of Environmental Sciences and Engineering, University of North Carolina, Chapel Hill, NC 27599. ^11^Department of Veterinary Integrative Biosciences, Texas A&M University, College Station, Texas 77843, USA. ^12^Department of Genetic Medicine and Development, University of Geneva Medical School, 1211 Geneva, Switzerland. ^13^Institute for Genetics and Genomics in Geneva (iG3), University of Geneva, 1211 Geneva, Switzerland. ^14^Swiss Institute of Bioinformatics, 1211 Geneva, Switzerland. ^15^Department of Genetics, Stanford University, Stanford, California 94305, USA. ^16^New York Genome Center, New York, New York 10011, USA. ^17^Department of Systems Biology, Columbia University Medical Center, New York, New York 10032, USA. ^18^Wellcome Trust Centre for Human Genetics Research, Nuffield Department of Clinical Medicine, University of Oxford, Oxford, United Kingdom OX3 7BN. ^19^Department of Computer Science, Stanford University, Stanford, California 94305, USA. ^20^Department of Computer Science, Johns Hopkins University, Baltimore, Maryland 21218, USA. ^21^Centre for Genomic Regulation (CRG), Dr. Aiguader 88, 08003 Barcelona, Spain. ^22^Universitat Pompeu Fabra, 08003 Barcelona, Catalonia, Spain. ^23^Human Genetics Department, McGill University, H3A 0G1 Montréal Canada. ^24^National Institute for Scientific Computing, Petropolis 25651-075 Rio de Janeiro, Brazil. ^25^Department of Stem Cell and Regenerative Biology, Harvard University, Cambridge, Massachusetts 02138, USA. ^26^Universitat de Barcelona, 08028 Barcelona, Catalonia, Spain. ^27^Radboud University Nijmegen, Netherlands. ^28^Institut Hospital del Mar d'Investigacions Mèdiques (IMIM), 08003 Barcelona, Spain. ^29^Oxford Centre for Diabetes, Endocrinology and Metabolism, University of Oxford, Churchill Hospital, Oxford, United Kingdom OX3 7LJ. ^30^Oxford NIHR Biomedical Research Centre, Churchill Hospital, Oxford, United Kingdom OX3 7LJ. ^31^Section of Genetic Medicine, Department of Medicine and Department of Human Genetics, University of Chicago, Chicago, Illinois 60637. ^32^Department of Human Genetics, University of Chicago, Chicago, Illinois 60637, USA. ^33^INRA, Department of Plant Biology and Breeding, AGAP, Montpellier, 34060, France. ^34^Department of Biostatistics, University of Michigan, Ann Arbor, Michigan 48109, USA. ^35^Department of Statistics, University of Chicago, Chicago, Illinois 60637, USA. ^36^Department of Genetics and Biology, Stanford University, Stanford, California 94305, USA. ^37^Howard Hughes Medical Institute, Chicago, Illinois, USA. ^38^Department of Genetics and Genomic Sciences, Icahn School of Medicine at Mount Sinai, New York, New York 10029, USA. ^39^Icahn Institute of Genomics and Multiscale Biology, Icahn School of Medicine at Mount Sinai, New York, New York 10029, USA. ^40^Department of Statistics, Harvard University, Cambridge, Massachusetts 02138. ^41^National Disease Research Interchange, Philadelphia, Pennsylvania 19103, USA. ^42^Roswell Park Cancer Institute, Buffalo, New York 14263, USA. ^43^Science Care, Inc., Phoenix, Arizona, USA. ^44^Gift of Life Donor Program, Philadelphia, Pennsylvania 19103, USA. ^45^LifeNet Health, Richmond, Virginia 23227, USA. ^46^UNYTS, Buffalo, New York 14203, USA. ^47^Virginia Commonwealth University, Richmond, Virginia 23298, USA. ^48^Department of Public Health, Temple University, Philadelphia, Pennsylvania 19122, USA. ^49^Van Andel Research Institute, Grand Rapids, Michigan 49503. ^50^Biorepositories & Biospecimen Research Branch, National Cancer Institute, Bethesda, Maryland 20892, USA. ^51^National Institutes of Health, Bethesda, Maryland 20892, USA. ^52^Biospecimen Research Group, Clinical Research Directorate, Leidos Biomedical Research, Inc., Rockville, Maryland 20852, USA. ^53^Sapient Government Services, Arlington, Virginia 22201-2909. ^54^Brain Endowment Bank, Department of Neurology, Miller School of Medicine, University of Miami, Miami, Florida 33136, USA. ^55^Division of Genomic Medicine, National Human Genome Research Institute, Bethesda, Maryland 20892, USA. ^56^Division of Genomics and Society, National Human Genome Research Institute, Bethesda, Maryland 20892, USA. ^57^Office of Science Policy, Planning, and Communications, National Institute of Mental Health, Bethesda, Maryland 20892, USA. ^58^Division of Neuroscience and Basic Behavioral Science, National Institute of Mental Health, Bethesda, Maryland 20892, USA. ^59^Cancer Diagnosis Program, National Cancer Institute, Bethesda, Maryland 20892, USA.
